# Supplementary material for: Continuous Assessment of Mental Workload During Complex Human–Machine Interaction: Inferring Cognitive State from Signals External to the Operator
Source: Sensors (Basel). 2025 Jun 9;25(12):3624. doi: 10.3390/s25123624 (PMC12197284; doi:10.3390/s25123624)
Supplement: Supplementary file 1 [file sensors-25-03624-s001.zip › sensors-3553722-supplementary/Supplementary Materials - Features - Review.pdf]

Supplementary materials

Features computation

Physiology: heart rate and inter-beat interval. The heart rate and inter-beat interval (IBI) were computed from the ECG signal measured by the Equivital sensor. The locations of the R waves were identified with a peak detection function (find_peaks from the scipy Python package). This resulted in a set of timestamps $\{t_i,\ 0 \le i \le n_{R-R} - 1\}$, with n_{R-R} the number of R-R intervals. With this formalism, the instantaneous heart rate hr_i (in bpm) at timestamp t_i takes the form:

$$hr_i = \frac{60}{t_{i+1} - t_i}. \tag{1}$$

Similarly, the instantaneous IBI ibi_i (in ms) can be expressed as:

$$ibi_i = 1000 * (t_{i+1} - t_i). \tag{2}$$

Hence the following formulas for the mean heart rate and IBI and their unbiased standard deviation estimator:

$$\bar{\mathrm{m}}_{heart\ rate} = \frac{1}{n_{R-R} - 1} \sum_{i=0}^{n_{R-R}-2} hr_i, \tag{3}$$

$$\bar{m}_{ibi} = \frac{1}{n_{R-R} - 1} \sum_{i=0}^{n_{R-R}-2} ibi_i, \tag{4}$$

$$\sigma_{heart\ rate} = \sqrt{\frac{1}{n_{R-R} - 2} \sum_{i=0}^{n_{R-R}-2} (hr_i - \bar{m}_{heart\ rate})^2}\,, \tag{5}$$

$$\sigma_{ibi} = \sqrt{\frac{1}{n_{R-R} - 2} \sum_{i=0}^{n_{R-R}-2} (ibi_i - \bar{m}_{ibi})^2}. \tag{6}$$

Physiology: respiratory rate. The breathing rate was obtained directly from the Equivital sensor. Briefly, the sensor returned a time series containing the instantaneous breathing rate at different instants $\{t_i,\ 0 \le i \le n_{br} - 1\}$, with n_{br} the number of measurements in the time window considered. Let br_i denote the instantaneous respiratory rate at timestamp t_i. The mean and standard deviation of the breathing rate were computed with the following formulas:

$$m_{breathing\ rate} = \frac{1}{n_{br} - 1} \sum_{i=0}^{n_{br}-2} br_i, \tag{7}$$

$$\sigma_{breathing\ rate} = \sqrt{\frac{1}{n_{br} - 2} \sum_{i=0}^{n_{br}-2} \left(br_i - \bar{m}_{breathing\ rate}\right)^2}. \tag{8}$$

Physiology: eye movements. Saccades and fixations were identified in the raw eye trace using the traditional I-VT algorithm from Salvucci and Goldberg [78]. This basic yet powerful algorithm employs a velocity threshold to discriminate between fixation and saccadic sequences. Note that since eye movements were recorded in ecological conditions, the use of the terms saccades and fixations are not completely appropriate as the measured eye movements may in reality be the result of the overlap of multiple components of the oculomotor system. Here, we therefore loosely refer as saccades the fast ballistic movements of the eyes and as fixations the periods of relative absence of saccades.

The I-VT algorithm returns a set of n_f fixation sequences and a set of n_s saccadic sequences. Let $\mathcal{T}_e^i$ refer to the set of timestamps corresponding to the ith event e, with $e = f, s$ being either a fixation (index f) or a saccade (index s). The duration $\Delta\mathcal{T}_e^i$ of an event thus corresponds to the difference between its last and first timestamp. With the previous notations, the mean duration of fixations and saccades can easily be derived:

$$\bar{m}_{fixation\ duration} = \frac{1}{n_f}\sum_{i=0}^{n_f-1} \Delta\mathcal{T}_f^i, \quad (9)$$

$$\bar{m}_{saccade\ duration} = \frac{1}{n_s}\sum_{i=0}^{n_s-1} \Delta\mathcal{T}_s^i. \quad (10)$$

Let us denote by $\mathcal{I}^i$ the set of indices corresponding to the ith saccade. The amplitude of such saccade corresponds to the planar distance between its last $\mathcal{I}_e^i$ and first $\mathcal{I}_b^i$ samples, i.e.:

$$\bar{m}_{saccade\ amplitude} = \frac{1}{n_s}\sum_{i=0}^{n_s-1} d_i\left(\mathcal{I}_b^i, \mathcal{I}_e^i\right), \quad (11)$$

with d_i the Euclidian distance.

Physiology: gaze ellipse area. The gaze of participants was projected to a 2-dimensional plane. Its dispersion was quantified using the area of 95% confidence ellipse. The calculation of the area of the ellipse exceeds the scope of this article, but the interested reader can refer to [79] for more details. Here, we merely report the formula for its computation:

$$95\%_{confidence\ ellipse} \approx 2 * \pi \frac{n+1}{n}\frac{n-1}{n-2} F_{1-\alpha,2,n-2}^2 \sqrt{\det(S)}, \quad (11)$$

where n is the signal's length, $F_{1-\alpha,2,n-2}$ the 0.95-quantile of the Fischer distribution with 2 and $n-2$ degrees of freedom, and $\det(S)$ the determinant of the sample covariance matrix S.

Physiology: gaze position (AoI). The different flight instruments in the cockpit were precisely delimited into areas of interest (AoI). The time spent in each AoI (in s) was computed. More precisely, let us define by $\boldsymbol{\mathcal{AOI}}$ the set of all regions of interest. Let us further declare each $r \in \boldsymbol{\mathcal{AOI}}$ the set of finite cardinal $|r|$ of timestamp indices that correspond to when the gaze landed in that AoI. If we further define the mean inter-sample duration δt of the gaze signal, the proportion of time spent in each region takes the form:

$$time\ spent\ in\ r = \delta t|r|, \forall r. \quad (12)$$

Machine: helicopter position. Helicopter positional information was measured directly from the simulator as a time series. The mean value of the pitch and roll orientation of the helicopter, as well as the standard deviations of the altitude, yaw, pitch, and roll signals were computed using the usual formulas (such as the ones presented above) and thus will not be elaborated further. In order to model the rate of change in each of these signals, the number of mean crossings – i.e., the number of times a signal crosses its mean – was computed. This metric was designed to capture some sort of frequency information, the idea being that fast-varying signals will present a greater number of mean-crossings than slow-varying signals. Let $S = (S_i)_{0 \le n \le N-1}$, stand for one of the helicopter position signal – namely the altitude, or the yaw, the pitch or the roll orientation – of length N. Let us denote by $\hat{S}$ the mean-centered signal derived from S. The mean-crossing points of S correspond to the zero-crossing points of $\hat{S}$. We refer to the zero-crossing points as the set of indices $\mathcal{Z} = \left\{z_j \mid 0 \le j \le J-1, J \in \{0, \dots, N-1\}\right\}$. They verify:

$$S_{z_j - 1} S_{z_j} < 0, \forall j \in \{1, \dots, J-1\} \quad (13)$$

The number of mean-crossings is simply given by the cardinal of $\mathcal{Z}$:

$$number\ of\ mean\ crossings = |\mathcal{Z}| = J \quad (14)$$

Machine: automatic pilot. In helicopters, the automatic pilot (AP) declines into two main components: vertical and horizontal modes. Each mode is then subdivided into 8 sub-modes. AP usage was monitored. The two resulting signals consist of a succession of numerical values corresponding to these different sub-modes. Similarly as with the gaze position, let us define by $\boldsymbol{\mathcal{M}}^c$ the set of all sub-modes of the AP, with $c = v, h$ being the vertical (v) or horizontal (h) component. Let us further declare each $m^c \in \boldsymbol{\mathcal{M}}^c$ the set of finite cardinal $|m|$ of timestamp indices that correspond to when the pilot used the specific sub-mode. If we further define the mean inter-sample duration δt of the signal, the proportion of time spent in each sub-mode takes the form:

$$time\ spent\ in\ m = \delta t|m^c|, \forall m^c, c \in \{v, h\}. \quad (15)$$

Human-machine interface: radio communication. The proportion of time spent in communication (%) was inferred from the "push-to-talk" signal from the radio device. The duration during which the latter signal took the value 1 was divided by the total duration of the time window considered.

Human-machine interface: flight commands. Sensors from the simulator allowed for the precise quantification of the actions on the manipulanda. In particular, the mean displacement (in %) and its standard deviation, as well as the mean force applied and its standard deviation (in daN) were measured for the following instruments: the thrust lever, the anti-torque pedals, the cyclic pitch, and the cyclic roll. They were computed using the standard formulas presented above. For both the displacement and the force signals, and for all of the aforementioned instruments, the number of mean crossings was quantified, with the exact same formula given for the helicopter position.
