# Supplementary material for: Continuous Assessment of Mental Workload During Complex Human–Machine Interaction: Inferring Cognitive State from Signals External to the Operator
Source: Sensors (Basel). 2025 Jun 9;25(12):3624. doi: 10.3390/s25123624 (PMC12197284; doi:10.3390/s25123624)
Supplement: Supplementary file 1 [file sensors-25-03624-s001.zip › sensors-3553722-supplementary/Supplementary Materials - Figures - Review.pdf]

Supplementary materials

Full-Flight Simulator

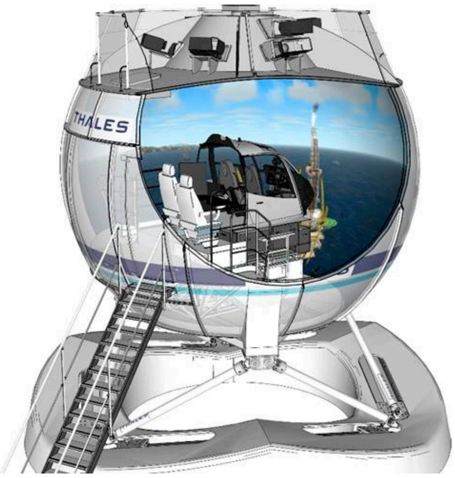

Figure S1: The EC135 cabin in the display dome, standing over the six-degrees-of-freedom motion platform. The pilot was seated in the right seat of the cabin.

Experimental setup

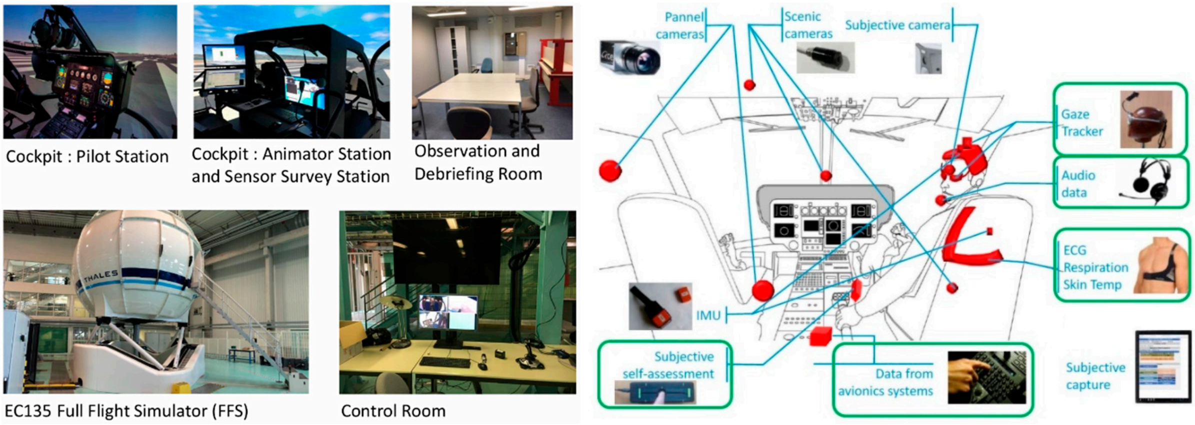

Figure S2: Left: Experimental environment. Right: the Crew Monitoring System® by Thales Equipment® records and synchronizes data from the sensors outlined in green. Additional sensors (cameras, inertial measurement units (IMU), etc.) were used to verify and validate the proper functioning of the sensors.

Weak classifier decision rule

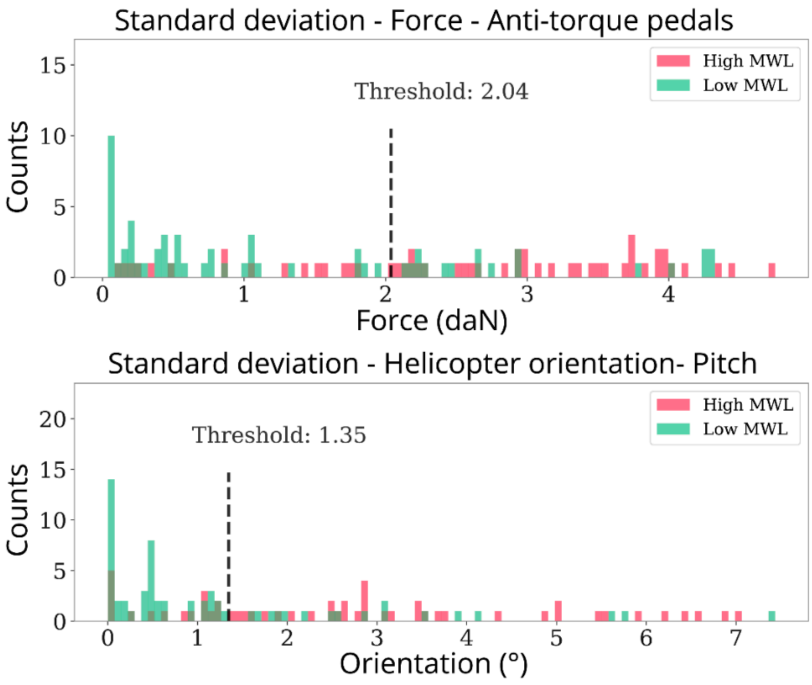

Figure S3: Two examples of the decision rules learned by the HBagging model on the complete dataset. The histograms represent the distribution of the values of two features (top: standard deviation of anti-torque pedals, below: standard deviation of pitch) over the set of time windows used for training the algorithm. The color of each bar indicates the MWL state associated with each time window which is known from the training dataset. In both examples, the features have been assumed a priori to increase with workload; therefore a threshold has been trained to classify low MWL states below the threshold and high MWL states above the threshold. Then, when the algorithm is used to classify new time windows with unknown workload labels, the algorithm classifies time windows with feature values above (respectively below) the threshold as high MWL (respectively low MWL). Note that the decision rule produces important errors even over the training set. It is the multimodal combination of several features that helps to increase performance.

Binarization of the MWL assessments

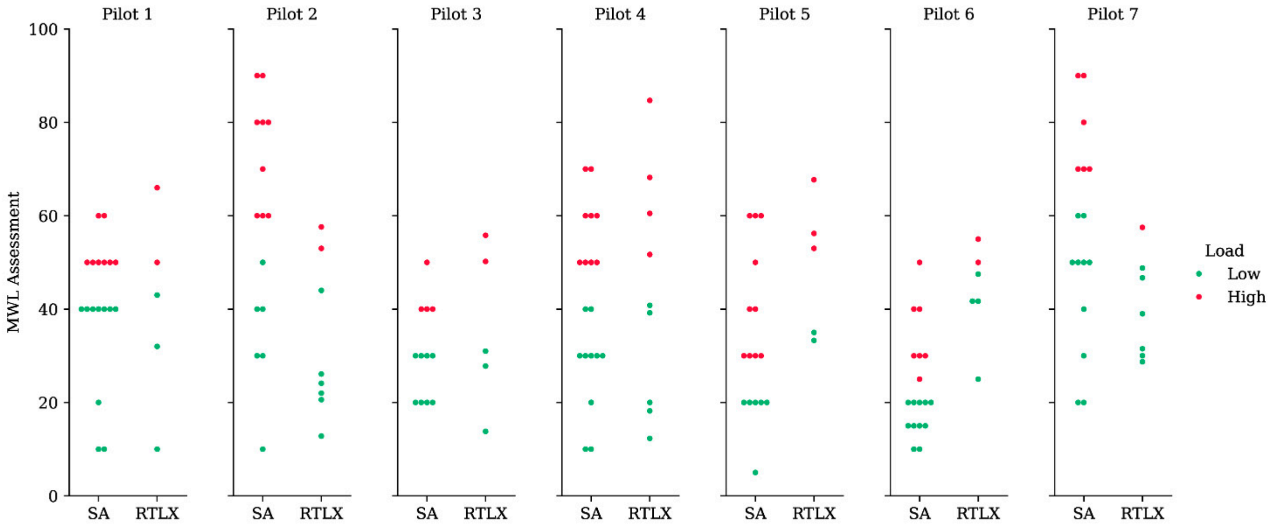

Figure S4: Swarm plot of the assessments of MWL for each pilot using two evaluation methods: SA (self-assessment) and RTLX. Green dots represent 'low' MWL values whereas red-colored dots are 'high' values, according to each evaluation method's binarization process.
