# Supplementary material for: Continuous Assessment of Mental Workload During Complex Human–Machine Interaction: Inferring Cognitive State from Signals External to the Operator
Source: Sensors (Basel). 2025 Jun 9;25(12):3624. doi: 10.3390/s25123624 (PMC12197284; doi:10.3390/s25123624)
Supplement: Supplementary file 1 [file sensors-25-03624-s001.zip › sensors-3553722-supplementary/Supplementary Materials - Tables - Review.pdf]

Supplementary materials

Extended best features table

Table S1: The best features returned by the model trained with the subjective oral declaration as ground truth and the complete dataset (1000 repetitions). Only features used over 2% of the time are shown.

Features	Percentage of use
Standard deviation of the displacement: pedals, cyclic in the pitch and roll planes	100, 100, 100
Standard deviation of the position of the helicopter: yaw, pitch and roll planes	100, 100, 100
Standard deviation of the force applied: pedals, cyclic in the pitch plane	100, 100
Movement frequency of the pedals	100
Proportion of time looking outside of the cockpit	100
Standard deviation of the displacement and of the force applied on the collective lever	98.7, 97.6
Proportion of time spent with a horizontal or vertical autopilot	98.5, 97.5
Standard deviation of helicopter altitude	95.7
Mean heart rate	93.3
Gaze ellipse area	89.0
Standard deviation of the force applied on the cyclic in the roll plane	84.2
Mean inter-beat interval	84.0
Proportion of time spent in communications	77.7
Frequency of helicopter movement in the yaw plane	55.4
Mean force applied on the cyclic in the roll plane	37.5
Mean displacement of the pedals	36.4
Mean displacement of the cyclic in the roll plane	26.9
Mean helicopter position in the pitch plane	19.9
Mean saccade duration	19.3
Movement frequency of the cyclic in the roll plane	18.6
Mean saccade amplitude	9.5
Mean force applied on the collective lever	5.0
Standard deviation of the heart rate	3.2
Frequency of helicopter movement in the pitch plane	2.1
